# Factors influencing breast cancer screening practices among women worldwide: a systematic review of observational and qualitative studies

**DOI:** 10.1186/s12905-024-03096-x

**Published:** 2024-04-27

**Authors:** Banafsheh Tavakoli, Awat Feizi, Fereshteh Zamani-Alavijeh, Hossein Shahnazi

**Affiliations:** 1 Student Research Committee, School of Health, Isfahan University of Medical Sciences, Isfahan, Iran; 2 Department of Epidemiology and Biostatistics, Isfahan University of Medical Sciences, Isfahan, Iran; 3 Department of Health Education and Health Promotion, School of Health, Isfahan University of Medical Sciences, Isfahan, Iran

**Keywords:** Breast Cancer, Screening behaviors, Cultural factors, Social Support, Systematic review

## Abstract

**Background:**

The variation in breast cancer incidence rates across different regions may reflect disparities in breast cancer screening (BCS) practices. Understanding the factors associated with these screening behaviors is crucial for identifying modifiable elements amenable to intervention. This systematic review aims to identify common factors influencing BCS behaviors among women globally.

**Methods:**

Relevant papers were sourced from PubMed, Scopus, Embase, and Google Scholar. The included studies were published in English in peer-reviewed journals from January 2000 to March 2023 and investigated factors associated with BCS behaviors.

**Results:**

From an initial pool of 625 articles, 34 studies (comprising 29 observational and 5 qualitative studies) with 36,043 participants were included. Factors influencing BCS behaviors were categorized into nine groups: socio-demographic factors, health status history, knowledge, perceptions, cultural factors, cues to action, motivation, self-efficacy, and social support. The quality appraisal scores of the studies ranged from average to high.

**Conclusions:**

This systematic review highlights factors pivotal for policy-making at various levels of breast cancer prevention and assists health promotion professionals in designing more effective interventions to enhance BCS practices among women.

**Supplementary Information:**

The online version contains supplementary material available at 10.1186/s12905-024-03096-x.

## Background

Breast cancer stands as the most commonly diagnosed cancer among women worldwide, affecting both developed and developing countries [1]. Statistical analyses indicate that while wealthier nations report higher breast cancer incidence rates, less developed countries suffer from higher relative mortality rates [2].

In high-income countries, including the United Kingdom, Australia, and Eastern Europe, over 60% of women are diagnosed at stages one and two of the disease, significantly improving their survival rates. Conversely, women in low-income countries often seek treatment at advanced disease stages when it has metastasized to other organs [3].

Differences in cancer incidence rates across populations may be attributable to the variance in risk factor prevalence and the implementation or uptake of screening programs [4].

Routine screening is pivotal in detecting breast cancer at an early, more treatable stage, significantly reducing mortality rates [5]. The primary methods of screening include breast self-examination (BSE), clinical breast examination (CBE) by a healthcare professional, and mammography (MMG), all of which have been demonstrated to lower mortality rates from breast cancer in various studies [6–9].

Despite numerous interventions and educational efforts aimed at promoting participation in BCS programs, recent studies indicate a continuing rise in mortality rates and a persistently low participation rate among women, particularly in less developed countries [1, 10]. For instance, recent figures show that only 13.6% of Malaysian, 0.3% of Egyptian, and 3.8% of Ethiopian women have undergone MMG in the past two years, compared to 81%, 88%, and 70% in Belgium, Australia, and the United States, respectively [11–16]. These disparities highlight the crucial need for developing and implementing effective strategies based on scientific and reliable research to enhance screening behaviors across different societies.

Given the significance of BCS and the dire predictions that both morbidity and mortality from breast cancer will more than double by 2035 [3], it becomes imperative to conduct a comprehensive review of the published literature. This systematic review aims to [1] summarize current knowledge on factors influencing BCS behaviors and [2] identify factors relevant to enhancing screening behaviors among women worldwide. Achieving these objectives and leveraging the findings of this research could empower policymakers, researchers, and health promotion professionals to devise more effective prevention policies and interventions, thereby improving BCS behaviors through well-informed strategies.

## Methods

This systematic review was registered with PROSPERO under the registration number CRD42023432810. The presentation of findings adheres to the PRISMA checklist standards (Additional file 1).

### Search Strategy

The research question, structured according to the PICOS framework, was: “What are the factors impacting BCS behaviors among women worldwide?”

The PICOS elements defined were as follows:

- Population: Healthy individuals aged 15 years or older, encompassing all genders, races, and geographic locations.
- Intervention (Influential Factors): This includes socio-demographic factors, health history, knowledge, perceptions, cultural factors, cues to action, motivation, self-care, and social support.
- Comparison Group: Subpopulations and subgroups differentiated by socio-demographic variables.
- Outcome: Practices related to BCS.
- Study Design: The review included cross-sectional, retrospective, prospective, and qualitative studies.

Four key search concepts and their synonyms (Table 1) were identified for the search. The international databases searched included PubMed, Scopus, Science Direct, Embase, and Google Scholar. Berenguer and Sakellariou’s search strategy [17] was adopted. The search concepts, along with their synonyms (utilizing truncations and wildcards, as indicated in Tables 1 and Additional file 2), where the asterisk ‘*’ was applied where appropriate, and subject heading terms were combined using the Boolean operators ‘OR’ within concepts, and ‘AND’ to combine concepts, thus developing the final search strategy (Additional file 2).

**Table 1:** Search key terms achieved from the research question

Factor	Associat*	Participate*	Breast cancer screening practices
Determin*	Relat*	Adherence*	Breast cancer screening behaviors
Predict*	Impact	Attendance*	Breast cancer screening programs
Barrier	Dependent	Uptake	Breast cancer prevent* programs
Enabler	Affect		Breast cancer screen*
Facilitator			Mammogra*
			Clinical breast exam*
			Breast self-exam*

*Some letters have been added to these words in the search time

### Inclusion and exclusion criteria

Studies were included if they:

1. Reported on MMG, CBE, or BSE as methods for BCS, in alignment with recommendations by international health organizations.
2. Were published in peer-reviewed journals between January 2000 and March 2023.
3. Addressed factors associated with BCS behaviors, focusing on associated factors rather than the effects of interventions.
4. Employed quantitative or qualitative research designs.
5. Included participants aged 15 years or older.

The exclusion criteria for the studies were:

1. Duplicate publications across databases.
2. Non-original research articles, including dissertations, reviews, case reports, editorials, oral and poster presentations, and book chapters.
3. Publications in languages other than English.
4. Preprints are not subjected to peer review.
5. Studies focusing on general cancer screening are not specific to breast cancer.
6. The research concentrated on other preventative behaviors or early detection methods unrelated to BCS.
7. Studies focused on factors associated with the second BCS participation round.
8. Research involving women with specific conditions, such as those who are sick or vulnerable.

### Study selection

The selection followed PRISMA guidelines. Initially, duplicates across databases were removed. Titles and abstracts were then reviewed for relevance, and articles not meeting the inclusion criteria were discarded. Subsequently, full texts of the remaining studies were evaluated for relevance, with any further non-compliant studies excluded. This review process was independently conducted by two researchers, with any discrepancies resolved through discussion.

### Quality assessment

Following numerous academics’ recommendations, the methodological quality of the included studies was assessed, and a Methodological Quality Score (MQS) was assigned. Experts evaluated each study’s conceptual and methodological rigor, resolving discrepancies by consensus. Based on Bernstein’s standards [18] and as explained by Patton [19], the assessment criteria included theoretical framework usage, study design, sample size, measurement instruments, data analysis, and reporting on reliability and validity. Quantitative studies were scored on a scale from 0 to 19, and qualitative studies from 0 to 14, with higher scores indicating higher methodological quality. Studies scoring below 60% were excluded.

### Data extraction and synthesis

Data were independently extracted by two researchers (BT and HSH), using a pre-designed tool to collect methodological details, including first author, publication year, study design, data source, study location, sampling strategy, sample size, data collection techniques, participant age, BCS method, and conceptual framework. For quantitative studies, additional data on screening participation rates and identified factors associated with BCS behaviors were noted. Qualitative studies included thematic information extracted for analysis.

## Results

**Fig. 1:**
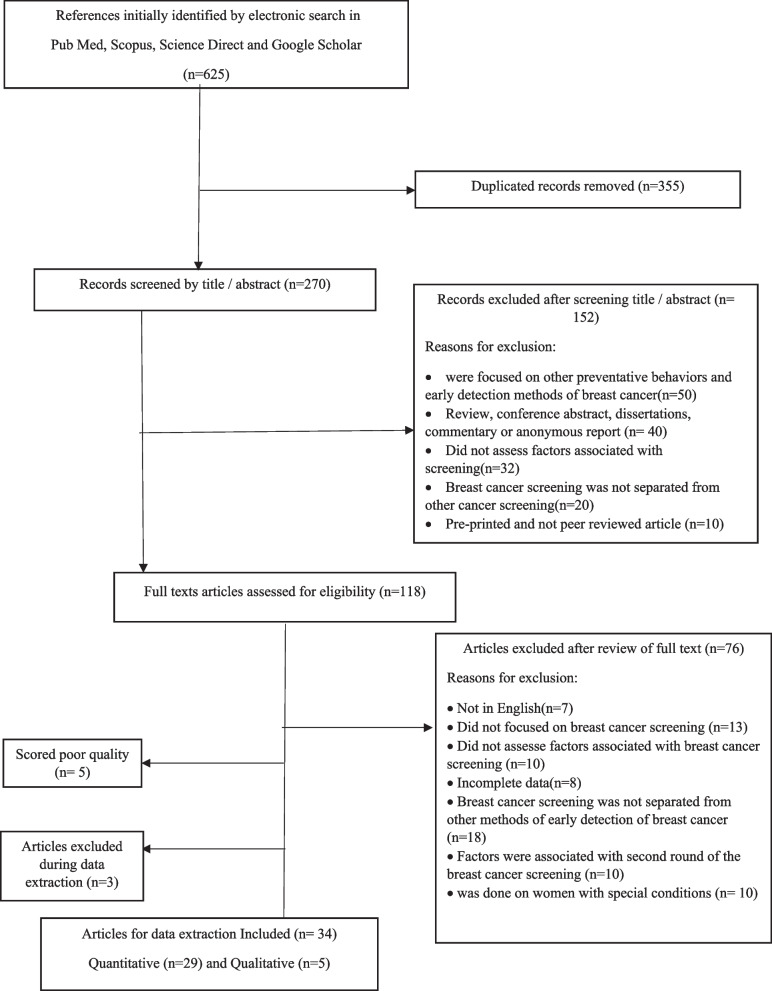
PRISMA flow diagram of the study selection procedure

An initial search yielded 625 articles from the specified databases. After removing duplicates and screening titles and abstracts, 118 papers were selected for full-text evaluation. Ultimately, 34 papers comprising 29 observational studies and 5 qualitative studies, with 36,043 participants, were included in the final review. The study selection process is illustrated in Fig. 1.

### Quality of included studies

None of the studies achieved the highest possible score. A majority of the studies were cross-sectional designs (82.4%), and over half (64.7%) included large samples (more than 300 participants). Furthermore, 67.7% of the studies grounded their findings in specific theoretical frameworks. Approximately half reported the psychometric properties of their assessment instruments. A significant portion (85.3%, *N* = 29) of the studies were quantitative and utilized both descriptive and advanced statistical analyses, such as t-tests, multiple regression, logistic regression, and multivariate analysis. The qualitative studies (14.7%, *N* = 5) primarily employed content and thematic analysis. All quantitative studies assessed the statistical significance of factors associated with BCS behaviors (Table 2).

**Table 2:** Criteria for methodological quality assessment of reviewed studies and the frequency distributions of each criterion

Methodological Characteristic	Scoring Options	Frequency (n)	Percent (%)
Theoretical Framework	The study had no theory = 0 points	11	32.3
	The study was based on a specific theory = 2 points	23	67.7
Design			
Study Design	Cross-sectional = 1 point	28	82.4
	Retrospective = 2 points	0	0
	Prospective = 3 points	1	2.9
	Qualitative = 3 point	5	14.7
Sample and measures			
Sample size	Small sample (< 100) = 1 point	8	23.5
	Medium sample (> 100 and < 300) = 2 points	4	11.8
	Large sample (> 300) = 3 points	22	64.7
Measuring Instrument	Not reported = 0 point	0	0
	Authors developed the instrument measuring factors = 1 point	15	44.1
	Authors adopted a previously established instrument = 2 points	19	55.9
Analytical approaches			
Data analysis	Univariate statistics/descriptive = 1 point	2	5.9
	Bivariate statistics/ANOVA = 2 points	1	2.9
	Multiple/logistic regression/ANCOVA = 3 points	24	70.6
	Qualitative analysis (content & thematic analysis) = 3points	5	14.7
	Multivariate statistics (structural equation modeling) = 4 points	2	5.9
Reliability	Not reported = 0 points	19	55.9
	Reported = 1 point	15	44.1
Validity	Not reported = 0 points	19	55.9
	Reported = 1 point	15	44.1
Results			
Factors Associated with BC Screening	No factors were identified = 0 points		
	Uncontrolled analysis (factors were not tested for statistical significance) = 1 point	5	14.7
	Controlled analysis (factors were tested for statistical significance) = 2 points	29	85.3
Conclusions	Not appropriate = 0 points		
	Appropriate = 1 point	34	100

### Characteristics of included studies

The 34 articles that met the inclusion and exclusion criteria were geographically diverse: 20 studies were conducted in Asia [10, 11, 20–37], 5 in America [16, 38–41], 4 in Europe [14, 42–44], 4 in Africa [12, 13, 45, 46], and 1 in Australia [15].

The sample sizes ranged from 8 to 11,409 participants, with the age of participants spanning from 15 to 82 years. Except for one qualitative study focusing on Arab men’s perceptions of female BCS [34], all participants were women.

There was variability in the BCS methods and the measurement of related factors across studies. Eleven studies identified CBE, BSE, or MMG as the screening methods [13, 20, 22, 29, 30, 32, 34, 36, 37, 41, 46]; four defined BSE or MMG [12, 25, 31, 35]; one mentioned CBE or MMG [11]; one mentioned CBE or BSE [24]; one specified CBE alone [39]; six identified BSE alone [23, 26, 28, 33, 45, 47]; and ten focused solely on MMG [14–16, 27, 38, 40, 42–44, 48].

The reported BCS rates varied significantly across studies, from 0.3 to 62% for BSE, 2.5–41% for CBE, and 0.3–88.1% for MMG (Table 3).

**Table 3:** Summary of the characteristics of included studies reviewed

Author, Year	Study design	Data source	Country	Sampling method	Sample size	Participants and age of participants	Screening method and Screening participation rate	Conceptual framework	MQS
Safarpour et al., 2018 [24]	Cross-sectional	Questionnaire	Iran	Simple Random	304	Women 20–65	BCS (BSE or CBE):17.1%	Knowledge-Attitude Practice Model	15
Moreira et al., 2018 [38]	Cross-sectional	Questionnaire	Brazil	Convenient	40	Women 50–69	MMG: Not Reported	Health Belief Model	12
Dewi et al., 2019 [33]	Cross-sectional	Questionnaire	Indonesia	Multistage, Stratified And Cluster, Random	1967	Women 20–60	BSE: 44.4%	Health Belief Model	15
Fouladi et al., 2013 [35]	Cross sectional	Questionnaire	Iran	Convenient	380	Women ≥ 30	BSE: 27% MMG: 6.8%	Health Belief Model	14
Canbulat and Uzun, 2008 [31]	Cross-sectional	Questionnaire	Turkey	Stratified And Systematic	268	Women ≥ 20	BSE: 21.9% MMG: 12.5%	Health Belief Model	14
Ahmad and Stewart, 2004 [39]	Cross-sectional	Questionnaire	Canada	Convenient	54	Women 25–60	CBE: 38.5%	Health Belief Model	11
Harirchi et al., 2012 [37]	Cross-sectional	Questionnaire	Iran	Stratified Simple-Random	770	Women ≥ 30	BSE: 36.6% CBE:17.4% MMG: 6.4%	Knowledge-Attitude Practice Model	13
Kardan-Souraki et al., 2019 [20]	Cross sectional	Questionnaire	Iran	Cluster	1165	Women ≥ 30	BSE: 62% CBE:41.1% MMG: 21.7%	None	13
Bailly et al., 2023 [44]	Cross-sectional	Statistical information units	France	Stratified Random	144	Women 50–74	MMG: 54-56%	None	11
Hajian-Tilaki and Auladi, 2014 [36]	Cross-sectional	Questionnaire Interview	Iran	Cluster	500	Women 18–65	BSE: 38.4 CBE:25.2% MMG: 12%	Health Belief Model	16
Tavafian et al., 2009 [28]	Cross-sectional	Questionnaire	Iran	Cluster	240	Women ≥ 30	BSE: 31.7%	Health Belief Model	15
Ahmadian et al., 2016 [47]	Cross-sectional	Questionnaire	Malaysia	Multistage Cluster Random	842	Women 17–52	BSE: NOT REPORTED	Health Belief Model	15
Jin et al., 2019 [16]	Cross-sectional	Questionnaire	USA	Purposive	303	Women 50–80	MMG: 73.3%	Andersen’s Behavioral Model	14
Charkazi et al., 2013 [32]	Cross-sectional	Questionnaire	Iran	Cluster	1080	Women 30–82	BSE: 13.1% CBE: 2.5% MMG:0.9%	Health Belief Model	13
Marmarà et al., 2017 [42]	Cross sectional	Questionnaire	Malta	Stratified Random	404	Women 50–60	MMG: NOT REPORTED	Health Belief Model & Common-Sense Model	16
Kangmennaang et al., 2019 [45]	Cross-sectional	Questionnaire Interview	Namibia	Cluster	9176	Women 15–64	BSE:35%	Health Belief Model	14
Secginli and Nahcivan, 2006 [25]	Cross-sectional	Questionnaire	Turkey	Convenience	656	Women ≥ 20	BSE: 17% MMG:25%	Health Belief Model	13
Racine et al., 2022 [41]	Cross-sectional	Questionnaire	Canada	Convenience	75	Women ≥ 18	BSE: 32% CBE: 12% MMG: 6.7%	Health Belief Model	14
Ma et al., 2012 [40]	Cross-sectional	Questionnaire	USA	Cluster Random & Proportional	682	Women ≥ 40	MMG: 50.04%	Sociocultural Health Behavior Model	13
Shakor et al., 2019 [26]	Cross-sectional	Questionnaire	Iraq	Non-Probability (Purposive)	750	Women ≥ 20	BSE: 18.0%	Health Belief Model	15
Thomas et al., 2011 [29]	Qualitative	Interview	Iran	Quota	31	Women 35–65	BCS: Not Reported	None	11
Hassan et al., 2017 [12]	Cross-sectional	Questionnaire Interview	Egypt	Systematic Random	600	Women ≥ 20	BSE: 0.3% MMG: 0.3%	None	12
Khazaee-pool et al., 2014 [48]	Qualitative	Interview	Iran	Purposive	16	Women ≥ 30	MMG: Not Reported	None	12
Moghaddam et al., 2019 [22]	Cross-sectional	Questionnaire	Iran	Multi-Stage Random	192	Women ≥ 30	BSE: 14% CBE:22.9% MMG: 10.1%	Pen-3 Model	14
Çam and Gümüs, 2009 [30]	Cross-sectional	Questionnaire	Turkey	Stratified Random	382	Women ≥ 40	BSE: 59.4% CBE:14.1% MMG: 34%	Health Belief Model	14
Moh Myint et al., 2020 [23]	Qualitative	Interview	Myanmar	Purposive	8	Women 20–45	BSE: Not Reported	None	11
Donnelly et al., 2017 [34]	Qualitative	Interview	Qatar	Purposive	50	Men 30–55	BCS: Not Reported	Ecological Perspective Klein Man’s Explanatory Model	13
Abeje et al., 2019 [13]	Cross-sectional	Questionnaire	Ethiopia	Multi-Stage Random	633	Women 20–49	BSE: 24.3% CBE:7.6% MMG: 3.8%	None	11
Carey and El-Zaemey, 2020 [15]	Cross-sectional	Questionnaire	Australia	Simple Random	1705	Women ≥ 40	MMG: 88.1%	None	11
Parsa and Kandiah, 2010 [11]	Cross-sectional	Questionnaire	Malaysia	Multi-Stage Random	425	Women 23–56	CBE:25% MMG: 13.6%	Health Belief Model	14
Tabrizi et al., 2018 [27]	Cross-sectional	Questionnaire	Iran	Multi-Stage Random	348	Women 30–60	MMG: 12%	None	12
Schoofs et al., 2017 [14]	Cross-sectional	Questionnaire	Belgium	Quota	350	Women 50–69	MMG: 81.5%	None	11
Lagerlund et al., 2015 [43]	cohort	Questionnaire	Sweden	Simple Random	11 409	Women 40–74	MMG: 88–95%	None	14
Elewonibi and BeLue, 2019 [46]	Qualitative	Interview	Nigeria	Convenience	94	Women ≥ 18	BCS: Not Reported	Pen-3 Model	12

*Abbreviations: BCS *Breast cancer screening, *BSE *Breast self-examination, *CBE *Clinical breast examination, *MMG *Mammography, *MQS *Methodological quality score

### Factors associated with BCS behaviors

The question of “What factors impact BCS behaviors in women worldwide?” is comprehensively answered through the analysis presented in Tables 4, 5 and 6. These tables delineate the factors influencing BSE, CBE, and MMG, respectively, as identified in the 34 reviewed articles.

The factors identified are categorized into nine key areas:

1. Socio-demographic Factors: This includes age, education level, income, marital status, and employment status, highlighting how these variables influence screening behaviors.
2. Health History: Past health experiences, family history of breast cancer, and personal health beliefs play a significant role in an individual’s decision to undergo screening.
3. Knowledge: The awareness and understanding of breast cancer and the benefits of early detection through screening methods.
4. Perceptions: Women’s beliefs and attitudes towards breast cancer risk, the effectiveness of screening, and the healthcare system’s role in cancer detection.
5. Cultural Factors: How cultural beliefs, norms, and societal expectations shape attitudes towards breast health and screening practices.
6. Cues to Action: External prompts, such as recommendations from healthcare professionals, health campaigns, or peers’ experiences, encourage women to seek screening.
7. Motivation: The intrinsic and extrinsic motivators drive women to participate in screening activities.
8. Self-care: The degree to which women prioritize their health and well-being, including the proactive pursuit of health screenings.
9. Social Support: The influence of family, friends, and community networks in supporting or hindering screening behaviors.

**Table 4:** Identified factors associated with breast self-examination behaviors among women around the world in the 34 reviewed articles

**Category**	**Factor**	**Breast self-examination behaviors**			
		**Studies displaying a positive association**	**Studies displaying a negative association**	**Studies displaying no association**	**Qualitative studies**
**Socio-demographic Factors**	Age	[20, 25, 32, 33, 45]		[24]	
	Education	[12, 13, 24–26, 32, 33, 45]	[37]		[34]
	Employment status	[13, 20, 26, 45]		[25]	
	Income	[13, 25, 45]			
	Marital status			[33]	
	Number of Children	[45]			
	Ethnicity	None			
	Region of Residence	[45]			
	Race	None			
	Spouse Demographic Characteristics	[13, 32]			
**Health History**	Hormone Therapy and History of Infertility	None			
	Family History of Breast Cancer	[13, 22, 26, 33]			
	Personal History of Cancer or Past Breast Disorders	[22, 26]			
**Knowledge **	Knowledge About Breast Cancer Screening	[12, 22, 24, 32, 37, 41]			[23, 29, 34, 46]
	Knowledge About Breast Cancer	[13, 22, 26]			[23, 46]
**Perceptions**	Perceived Health Status	[20]			
	Attitude Towards Breast Cancer Screening	[24, 37]			
	Perceived Barriers	[25, 26, 30, 33–35, 45, 47]		[36]	[23, 34]
	Perceived Benefits	[26, 28, 30, 31, 33, 36]			
	Self-efficacy	[10, 25, 26, 28, 31, 33, 35, 36]			[29]
	Perceived Severity	[26, 41]		[28, 32, 33, 35, 36]	[46]
	Perceived Susceptibility	[25, 32]		[28, 33, 36]	
**Cultural Factors**	Fatalistic /Religious Beliefs		[32]		[46]
	Cultural Differences) Longer Migration Time- speaking English Well- Cultural Support)				[29]
	Gender of the Doctor Performing the Clinical Checkup/Examinations				[34]
	Traditional/Alternative Care				[46]
	Social Stigma				[34]
**Cues to Action**	Breast Cancer Screening by Family Members and Friends	None			
	Hearing About BC and BCS from Health Team or in the Media	[22, 26]	[33]		
	Similarly, Reminder Letters, Phone Calls, or Text Messages	None			
**Motivation**	High Level of Hope and Health Motivation for the Future	[26, 30, 31, 36]			[29]
**Self-Care**	Having Regular Checkups	[45]			
	Smoking	[26]			
	Alcohol Abstinence	None			
	Physical Activity	None			
	Following Healthy Diet	None			
	Body Mass Index			[20]	
**Social Support**	Health Insurance Coverage	[25, 45]		[22]	
	Health Workers and Family Members Support			[22]	[29, 34, 46]
	Access to Screening Centers			[22]	[46]

**Table 5:** Identified factors associated with clinical breast examination behaviors among women around the world in the 34 reviewed articles

**Category**	**Factor**	Clinical breast examination behaviors			
		**Studies displaying a positive association**	**Studies displaying a negative association**	**Studies displaying no association**	**Qualitative studies**
**Socio-demographic Factors**	Age	[11, 32, 39]		[24]	
	Education	[13, 24, 32]	[37]		[34]
	Employment status	[13, 20]			
	Income	[11, 13, 20]			
	Marital status	None			
	Number of Children	None			
	Ethnicity	None			
	Region of Residence	None			
	Race	None			
	Spouse Demographic Characteristics	[13]		[20]	
**Health History**	Hormone Therapy and History of Infertility	[20]			
	Family History of Breast Cancer	[13, 22]		[11]	
	Personal History of Cancer or Past Breast Disorders	[11, 22]			
**Knowledge **	Knowledge About Breast Cancer Screening	[22, 24, 29, 32, 39, 46]			[29, 34, 46]
	Knowledge About Breast Cancer	[13, 22]			[46]
**Perceptions**	Perceived Health Status	None			
	Attitude Towards Breast Cancer Screening	[24, 37]			
	Perceived Barriers	[37, 39]		[36]	[34]
	Perceived Benefits	[11, 36, 41]			
	Self-efficacy	[36]			[29]
	Perceived Severity			[32, 36]	[46]
	Perceived Susceptibility	[11, 32]		[36]	
**Cultural Factors**	Fatalistic /Religious Beliefs	[41]	[32]		[46]
	Cultural Differences) Longer Migration Time- speaking English Well- Cultural Support)	[39]			[29]
	Gender of the Doctor Performing the Clinical Checkup/Examinations				[34]
	Traditional/Alternative Care				[46]
	Social Stigma				[34]
**Cues to Action**	Breast Cancer Screening by Family Members and Friends	None			
	Hearing About BC and BCS from Health Team or in the Media	[22]			
	Similarly, Reminder Letters, Phone Calls, or Text Messages	None			
**Motivation**	High Level of Hope and Health Motivation for the Future	[30, 36]			[29]
**Self-Care**	Having Regular Checkups	[11, 39]			
	Smoking	None			
	Alcohol Abstinence	None			
	Physical Activity	None			
	Following Healthy Diet	None			
	Body Mass Index			[20]	
**Social Support**	Health Insurance Coverage			[11, 22]	
	Health Workers and Family Members Support	[22]			[29, 34, 46]
	Access to Screening Centers			[22]	[46]

**Table 6:** Identified factors associated with mammography behaviors among women around the world in the 34 reviewed articles

Category	Factor	Mammography behaviors			
		Studies displaying a positive association	Studies displaying a negative association	Studies displaying no association	Qualitative studies
**Socio-demographic Factors**	Age	[15, 38, 41]	[14]		
	Education	[12, 13, 25, 38, 41]	[37]	[27]	[34]
	employment status	[13, 15]	[44]	[25, 27, 38]	
	Income	[13, 38, 42]		[11, 25]	
	Marital status	[38, 44]		[11, 27]	
	Number of Children	[15, 20, 38]		[27]	
	Ethnicity	[11, 40]			
	Region of Residence			[27]	
	Race			[38]	
	Spouse Demographic Characteristics	[13, 20]			
**Health History**	Hormone Therapy and History of Infertility	[15]			
	Family History of Breast Cancer	[13, 15, 22, 27, 38, 44]		[11]	
	Personal History of Cancer or Past Breast Disorders	[11, 16, 22, 38]		[27]	
**Knowledge**	Knowledge About Breast Cancer Screening	[12, 22, 27, 32, 41]			[29, 34, 46, 48]
	Knowledge About Breast Cancer	[13, 16, 22, 27]			[46]
**Perceptions**	Perceived Health Status	[11, 43]			
	Attitude Towards Breast Cancer Screening	[37]			
	Perceived Barriers	[30, 35, 37, 38, 41, 42]		[36]	[34]
	Perceived Benefits	[25]			
	Self-efficacy	[16, 42]			[29]
	Perceived Severity	[25]		[32, 35, 36]	[46]
	Perceived Susceptibility	[11, 25, 31, 32, 41]		[36]	
**Cultural Factors**	Fatalistic /Religious Beliefs	[41]	[32]		[46, 48]
	Cultural Differences) Longer Migration Time- speaking English Well- Cultural Support)	[40]			[29]
	Gender of the Doctor Performing the Clinical Checkup/Examinations				[34]
	Traditional/Alternative Care				[46]
	Social Stigma and Anticipated Negative	None			
**Cues to Action**	Breast Cancer Screening by Family Members and Friends	[40, 42]			
	Hearing About BC and BCS from Health Team or in the Media	[22, 27, 42]		[11]	
	Similarly, Reminder Letters, Phone Calls, or Text Messages	[42]			
**Motivation**	High Level of Hope and Health Motivation for the Future	[30]			[29, 48]
**Self-Care**	Having Regular Checkups	[11, 16, 25, 40]			
	Smoking	[20]	[43]		
	Alcohol Abstinence		[43]	[15]	
	Physical Activity		[43]		
	Following Healthy Diet		[14, 43]		
	Body Mass Index		[14]	[15, 20]	
**Social Support**	Health Insurance Coverage	[16, 25, 40]		[11, 22]	
	Health Workers and Family Members Support	[16, 22, 42]			[29, 34, 46, 48]
	Access to Screening Centers	[27, 40, 44]		[22]	[46]

## Discussion

The primary goal of this study was to identify the universal factors influencing BCS behaviors among women globally. Although most countries offer BCS programs [17], the nature and implementation of these programs vary significantly across different health systems and populations [49]. Consequently, the BCS methods examined in this review varied, reflecting these disparities. MMG, recognized for its efficacy in clinical studies, is predominantly used in developed countries due to its higher costs [8]. Conversely, in developing countries, BSE stands out as a widely adopted, cost-effective method for early detection [50].

Moreover, the rates of screening methods reported in the literature show considerable international variation. Countries like Sweden, Belgium, the USA, and Australia report high MMG screening rates [14–16, 43], whereas BSE is more prevalent in countries like Egypt, Ethiopia, Turkey, Iran, and Iraq [12, 13, 25, 26, 32], often falling below the WHO’s recommended screening rates [49].

The WHO underscores the importance of high participation rates in screening programs to enhance their effectiveness [49]. Understanding the factors influencing participation enables health systems to adopt comprehensive strategies for prevention, early diagnosis, and BCS promotion.

Over half of the studies reviewed focused on socio-demographic factors as determinants of screening behaviors, identified in previous research as facilitators and barriers [51, 52]. Findings indicate that demographic variables such as age, education level, income, and employment status significantly influence screening rates.

While socio-demographic status is recognized as a crucial determinant of access to BCS in both high-income [51, 52] and middle-income countries [10, 17], studies in European countries with organized screening programs report no correlation between screening participation and socio-demographic variables [53]. A 2011 study exploring the impact of socioeconomic inequalities on screening participation highlighted that such disparities exist even without financial barriers [54]. These variations necessitate careful interpretation, considering women’s diverse challenges in accessing screening services worldwide, including geographical, economic, and cultural obstacles.

For instance, despite Qatar’s provision of comprehensive medical services at no cost, including BCS, cultural barriers have led to only a third of eligible women utilizing these services [34]. Thus, offering organized screening programs with equitable access could gradually mitigate socioeconomic disparities.

The review also highlights that beyond a family history of breast cancer and personal breast health issues, fertility-related challenges, such as infertility and hormonal imbalances, influence screening behaviors. This finding aligns with systematic reviews from China and the USA, which examined screening factors among different populations [55, 56]. Women with personal or familial health histories may perceive a higher susceptibility to breast cancer, thereby increasing their utilization of healthcare services for screening and diagnostic tests. This heightened awareness and concern about breast cancer risk can motivate women to adopt preventive measures, including screening. However, it is notable that many women may not pursue screening until symptomatic or following the discovery of breast cancer in close relatives [57, 58].

The findings of the study reveal that women with comprehensive knowledge about breast cancer risk factors, symptoms, and screening methods are more likely to participate in screening programs. Conversely, women who have not undergone screening often lack awareness or believe that once screened, repeat screenings are unnecessary [59]. This lack of knowledge has been identified as a critical barrier to screening participation among Iranian and Asian women and as a predictive factor for the late diagnosis of breast cancer in Canada [10, 60, 61]. However, Schlueter’s study found no correlation between the level of knowledge and screening behaviors [62], indicating the complexity of this relationship.

Educational interventions targeting breast cancer awareness and screening guidelines are crucial for improving women’s knowledge and participation rates.

Perceptual factors significantly influence screening behaviors, including fewer perceived barriers and higher self-efficacy. A Chinese study highlighted reduced perceived barriers as a predictive factor for screening participation [55]. Main barriers identified include fear [34, 42, 46, 48], anxiety [29, 30], worry [22, 63], religious beliefs and fatalism [32, 46, 48], financial constraints [34], language barriers [29, 39, 40], and embarrassment [63]. Although fear can motivate screening behavior in some contexts [56], it is predominantly an emotional barrier in the findings.

Types of fear recognized include the fear of mastectomy, diagnosis of cancer, and stigmatization [34, 46, 48]. Consedine et al. noted that while fear of cancer could facilitate screening, specific fears—such as those associated with medical procedures or diagnosis—often deter women from participating [64]. A meta-analysis further linked fear of breast cancer to screening behaviors [65], suggesting that mitigating fear through education and positive screening experiences could enhance participation rates.

Cultural factors, particularly religious beliefs, and fatalism, notably impact screening behaviors. Some Muslim women believe BCS is unnecessary, viewing cancer as a divine challenge or part of destiny [63]. This fatalistic view, a belief in the health locus of control being external (chance or divine will), can lead to passive health behaviors [66]. While some studies show no significant impact of religious beliefs on screening behaviors [67], the intertwined nature of these beliefs with culture and religion necessitates nuanced interventions.

Effective strategies might involve integrating breast cancer awareness and early diagnosis information within the framework of existing belief systems leveraging religious leaders to promote health messages aligned with spiritual teachings. Such approaches, using religious and spiritual elements in health messaging, have been shown to encourage screening behaviors among women [11].

The results of this review highlight that women are more likely to engage in BCS behaviors when they receive information from healthcare teams, social media, or other sources compared to those who do not consult with healthcare professionals or use social media for health information. Jones et al. emphasized that recommendations and reminders from healthcare providers are among the most effective means of directing women toward MMG and other screening tests [68]. A 2019 study further showed that ignoring cues to action, such as letters, messages, and reminder calls, correlates with lower MMG participation rates [69].

In the modern era, widespread access to information through digital media, advancements in technology, and the introduction of electronic health tools have facilitated the use of these platforms in cancer screening campaigns. For instance, smartphone applications that remind users about screening schedules and provide preventive advice through text, images, and videos represent an innovative approach to enhancing screening participation.

This review also underscores a significant link between motivation and BCS behaviors. Khazaee-Pool et al. found that motivational solid factors, such as valuing life and health responsibility, significantly encourage screening participation [21]. Moreover, studies among diverse racial and ethnic groups have identified a clear association between motivation and increased screening activities [70].

Various socio-psychological barriers, including attitudes, cultural beliefs, and communication issues, have been identified as impediments to motivation [71]. Factors contributing to low motivation for MMG include the perceived unimportance of testing, lack of support, time constraints, cost concerns, familial obligations, and a busy lifestyle [48]. Therefore, interventions aimed at enhancing motivational self-efficacy could significantly improve screening participation.

As part of self-care practices, regular health check-ups have been shown to predict screening behaviors. Reviews have highlighted a correlation between infrequent mammograms and breast exams among Asian and Korean-American women with irregular gynecological visits [51, 59]. Although MMG can be performed without direct referrals in some countries [59], the lack of commitment to regular check-ups remains a barrier. As Pasket et al. reported, while 75% of women acknowledged the importance of periodic exams, 67% indicated that their physicians did not actively encourage MMG [72].

Improving knowledge about self-care and self-regulation is crucial for fostering regular health examination habits. The health system’s role in scheduling periodic health assessments and encouraging adherence is also vital, as demonstrated by research from the Netherlands, which linked pre-scheduled appointments and proactive general practitioner involvement to higher screening rates [49].

Regarding social support, assistance from healthcare teams and family members significantly influences screening behaviors. Lack of partner support and fear of familial disruption post-diagnosis have been noted as significant barriers among African-American women [68]. Support from family and friends, providing both financial and emotional backing, can bolster confidence, reduce fear, and encourage screening participation [21, 59, 73].

The review also points out that women’s financial independence and employment status in certain regions are critical in health decision-making. Conversely, many women rely on male family members to make health decisions, a group that requires targeted support from health teams for emotional, instrumental, and informational needs. Leong et al. found that social support not only reduces depression but also promotes healthier behaviors [74]. Thus, establishing support networks and self-help groups can enhance women’s knowledge, experience, and motivation regarding BCS, ultimately fostering a community of mutual encouragement and support.

### Strengths

This systematic review meticulously evaluated the quality of included studies to ensure their reliability and relevance. A unique aspect of the analysis is the consideration of men’s attitudes and perceptions toward BCS, acknowledging the influence of gender dynamics on screening behaviors. A comprehensive approach was undertaken, analyzing factors affecting BCS behaviors across quantitative and qualitative studies and categorizing them based on their impact on three distinct screening behaviors: BSE, CBE, and MMG. This nuanced categorization provides a detailed understanding of the diverse influences on BCS practices.

### Limitations

This research was confined to online studies, potentially overlooking valuable research indexed in databases such as PubMed, Scopus, Embase, and Google Scholar or available only in print. The restriction to English-language publications may have excluded pertinent non-English studies, introducing language bias. The review’s predominance of cross-sectional studies limits the ability to ascertain causal relationships between the factors studied and screening behaviors. Additionally, the reliance on self-reported data raises concerns about the accuracy of the findings, given the potential for recall bias or the desire of participants to present themselves in a socially desirable light.

The heterogeneity of the included studies—in terms of study design, geographic location, methodological approach, demographic characteristics, sample size, screening methods employed, and the intervals between screenings—complicates direct comparisons and may affect the generalizability of the findings.

## Conclusion

This systematic review synthesizes a broad array of research on the factors influencing BCS behaviors among women worldwide. By examining various screening methods and participation rates, along with identifying determinants of screening behavior, this study contributes valuable insights to the field of public health. The findings highlight the complex interplay of factors affecting screening behaviors and provide evidence-based guidance for policymakers and health promotion professionals. This knowledge is crucial for developing targeted interventions that can effectively encourage BCS practices, ultimately contributing to breast cancer prevention and early detection.

### Supplementary Information

**Supplementary Material 1.**

**Supplementary Material 2.**

## Data Availability

This published article and its supplementary information files include all data generated or analyzed during this study.

## Abbreviations

BCS: Breast cancer screening
BSE: Breast self-examination
CBE: Clinical breast examination
MMG: Mammography
MQS: Methodological quality score
PRISMA: Preferred Reporting Items for Systematic Reviews and Meta-Analyses
WHO: World Health Organization
