# Supplementary material for: Factors influencing breast cancer screening practices among women worldwide: a systematic review of observational and qualitative studies
Source: BMC Womens Health. 2024 Apr 27;24:268. doi: 10.1186/s12905-024-03096-x (PMC11055241; doi:10.1186/s12905-024-03096-x)
Supplement: Supplementary file 2 — Supplementary Material 2. [file 12905_2024_3096_MOESM2_ESM.docx]

Additional file 2:

**Table 2.1.** Combination of search terms used in the final search strategy for the literature review.

|  | **Combination of Search Terms** |
| --- | --- |
| 1 | Factor OR Determine* OR Predict* OR Barrier OR Enabler OR Facilitator |
| 2 | Exp Factor/ |
| 3 | 1 OR 2 |
| 4 | Associat* OR Relat* OR Impact OR Dependent OR Affect |
| 5 | Exp Associat* / |
| 6 | 4 OR 5 |
| 7 | Participate*OR Adherence*OR Attendance*OR Uptake |
| 8 | Exp Participate*/ |
| 9 | 7 OR 8 |
| 10 | Breast cancer screening behavior OR Breast cancer screening practices OR Breast cancer screening programs OR Breast cancer prevent* programs OR Breast cancer screen* OR Mammogra* OR Clinical breast exam* OR Breast self-exam* |
| 11 | Exp breast cancer screening behavior/ |
| 12 | 10 OR 11 |
| 13 | 3 AND 6 AND 9 AND 12 |
| 14 | Only articles published from 2000 until 2020 in English |
